# The Characteristics Of Human Bone-Derived Cells (HBDCS) during osteogenesis in vitro

**DOI:** 10.1186/s11658-016-0027-8

**Published:** 2016-11-16

**Authors:** Edyta Wrobel, Joanna Leszczynska, Edyta Brzoska

**Affiliations:** 1 Department of Biophysics and Human Physiology, Faculty of Health Sciences, Medical University of Warsaw, ul. Chalubinskiego 5, 02-004, Warsaw, Poland; 2 Department of Cytology, Institute of Zoology, Faculty of Biology, University of Warsaw, ul. Miecznikowa 1, 02-096, Warsaw, Poland

**Keywords:** Human bone-derived cells, Integrins, Osteoblast differentiation, Osteogenesis in vitro, Osteogenic markers, Primary osteoblasts

## Abstract

**Background:**

The primary human bone-derived cell culture technique is used as a model to study human osteogenesis. Compared to cell line cultures, primary osteoprogenitor and osteoblast cultures provide more complex information about osteogenesis, bone remodeling and regeneration than cell line cultures.

**Methods:**

In this study, we isolated human bone-derived cells (HBDCs) and promoted their differentiation into osteoblasts. The following parameters were evaluated: cell number and viability, total protein expression, alkaline phosphatase activity, collagenous matrix production and osteogenic genes expression, i.e., gene coding for type I collagen and alkaline phosphatase.

**Results:**

It was proved the results show that HBDCs intensively proliferate during the first 7 days of culture followed by differentiation accompanied by an increase in alkaline phosphatase activity. Moreover, it was observed that during the differentiation of HBDCs, the expression of integrin β1 increased.

**Conclusions:**

The process was also accompanied by changes in cell shape and rearrangement of the actin cytoskeleton and focal contacts containing FAK and the integrin β1 subunit. We suggest that the β1 integrin subunit may be a suitable new target in studies of the differentiation of primary human osteoblasts in culture.

## Background

Bone is a highly organized structure of calcified connective tissue formed during osteoprogenitor proliferation and differentiation into mature osteoblasts. Osteoblasts (bone-forming cells) are identified on the basis of morphology – their cuboidal appearance – and their association with bone matrix.

Osteoblast maturation consists of three main phases: proliferation, extracellular matrix (ECM) synthesis, and mineralization [1]. Osteoblast differentiation from their undifferentiated to functional state is accompanied by changes in cell morphology and in the expression of adhesion molecules, ECM proteins (collagen type I; COLI) and specific osteogenic markers, i.e., alkaline phosphatase (ALP), osteocalcin (OC), osteopontin (OP), osteonectin (ON) and bone sialoprotein (BSP). ALP is considered a relative early marker of osteoblast differentiation [2]. The subsequent reconstruction of a collagenous matrix occurs in the course of procollagen I mRNA decrease [3] and OC expression increase, a late marker of differentiated osteoblasts [1].

Osteoblast studies are performed using various culture models, such as immortalized cell lines (e.g., MG-63 or SaOS-2), induced osteoblasts from pluripotent stem cells, and harvested primary osteoblasts [4, 5]. The potential of in vitro cultured osteoblasts to maintain their phenotype and activity depends on the cell type used. In the case of primary osteoblasts, cell phenotype and potential depends on the age, site of isolation, donor gender and method of cell isolation and culture [5, 6]. The primary osteoblast culture could be obtained in the process of progressive enzymatic digestion of bone tissue [7] or cell migration from the bone explants [6]. Generally, using such a factor as ascorbic acid, 1α,25-dihydroxyvitamin D_3_ and dexamethasone, it is possible to induce osteogenesis of stem or progenitor cells, such as mesenchymal stem cells or induced pluripotent stem cells, or of osteoprogenitors and osteoblasts [5, 8–11]. In this study, we concentrated on the osteogenic potential of human bone-derived cells (HBDCs) obtained from bone explant enzymatic digestion and cultured in vitro.

Human osteogenic cells derived from various bone compartments are routinely established in short-term cultures. These cultures are heterogeneous and consist of different bone cell subpopulations. The subpopulations seem to be primary osteoprogenitor, premature and mature osteoblasts at various stages of differentiation [12]. Moreover, it was also described that in vitro HBDCs were able to differentiate into multiple mesenchymal lineages, suggesting their multipotent character [13, 14]. Importantly, the primary osteoblast cultures provide more complex information about osteogensis, bone remodeling and regeneration than cell line cultures. The differences between primary osteoblasts and cell line cultures should be taken into account when studying processes of osteoblast differentiation in vitro. The growth and differentiation of HBDCs is regulated by growth factors, cytokines and hormones, and also depends on the cell density in culture [5]. In this study, we focused on the role of cell adhesion in HBDC differentiation.

Cell adhesion supports tissue morphogenesis integrity as well as tissue repair. Bone cell adhesion to the extracellular matrix directly regulates cell growth, the expression of the osteoblast phenotype and the process of bone tissue formation [15].

Integrins are the adhesion molecules involved in the process of cell adhesion. Integrin-mediated adhesion is a highly regulated process involving receptor–ligand interactions and cell spreading. Upon ligand binding, integrins rapidly associate with the actin cytoskeleton and cluster together to form focal contacts, which are complexes composed of structural and signaling molecules such as focal adhesion kinase (FAK).

Focal adhesions act as structural links between the cytoskeleton and extracellular matrix and mediate stable cell adhesion and migration [16]. The binding of transmembrane integrin adhesion receptors to ECM components, such as fibronectin and COLI, activates signaling pathways engaged in cell-cycle progression, gene expression, matrix mineralization and survival of primary osteoblasts [17]. Osteoprogenitor cells and osteoblasts express multiple integrins, including α1β1, α2β1, α3β1, α4β1, α5β1, α8β1 and αvβ3, which bind to numerous ECM proteins [18]. Blocking adhesion with COLI-specific peptides or antibodies directed against the collagen-binding integrin α2β1 affects with the activity of Runx/Cbfa1 transcription factors, expression of osteoblast-specific genes, and matrix mineralization [19].

The purpose of this study was to characterize HBDC phenotypes by defining cell morphology changes and the proliferation and differentiation rate. We also analyzed actin cytoskeleton organization and adhesive protein expression. The phonotype of in vitro cell culture depends on the manipulation of the biological material and the culture conditions, including medium, time and the presence of compounds that influence cell proliferation and differentiation [20]. The primary focus of this study was HBDC biology in vitro as part of an evaluation of their tissue engineering potential.

## Methods

The Local Ethics Committee of the Warsaw Medical University approved all procedures (Decision No. KB/74/2005). All of the donors provided informed consent.

### Primary culture of HBDCs

Human bone-derived cells (HBDCs) were isolated from trabecular bone tissue chips harvested from the bottom distal part of the long tight bone during standard procedure knee joint alloplasty. Material from seven female patients ranging in age from 59 to 63 was used. Cells harvested from different donors were cultured separately. The procedure of HBDC isolation was based on the protocols described by Gallagher et al. [21] with modifications [22].

Briefly, the extracted bone pieces were purified from other tissues by scraping, and the bone was cut into 1-mm pieces and washed with phosphate-buffered saline (PBS) containing Ca^2+^ and Mg^2+^ ions (Gibco BRL, Life Technologies B.V. Breda, The Netherlands),.), and subsequently digested overnight using a collagenase XI S (200 U ml^−1^; Sigma-Aldrich, St. Louis, USA).).

Next, samples were washed with PBS and placed in a 75-cm^3^ cell culture flasks (Costar-Nunc), containing the standard culture medium: Dulbecco’s modified Eagle’s medium (DMEM; Gibco) supplemented with a 10% final concentration of heat-inactivated fetal bovine serum (FBS; Gibco), _L_-glutamine (Gibco), a 1% antibiotic–antimycotic mixture consisting of 10,000 U of penicillin (base), 10 mg of streptomycin (base) and 25 μg of amphotericin B ml^−1^ (Gibco), and 100 mM _L_-ascorbic acid 2-phosphate hydrate sesquimagnesium salt (Sigma-Aldrich).

The explants were cultured in a CO_2_ incubator at 37 °C in 5% CO_2_ and humidified atmosphere. The medium was changed every 7 days. Within 2–4 weeks, osteoblast migration from the bone explants was observed. Then the explants were placed in two or three different flasks to obtain two populations of cells originating from the same donor. After confluence, HBDCs were detached using collagenase and trypsin/EGTA (Gibco) treatment.

The cells from the first passage were used in all of the experiments. Cells were seeded in 24-well dishes at a density of 4 × 10^4^ cells per well (1.9 cm^2^) in a differentiating medium containing 10 nM dexametasone (Sigma-Aldrich) and 100 nM 1α,25-dihydrixycholecalciferol (Sigma-Aldrich) to induce osteogenesis. The morphology of the cells was analyzed using an inverted, phase-contrast microscope (Nikon Eclipse TE2000-u).

### Evaluation of cell proliferation and viability

To evaluate cell proliferation, HBDCs were plated at a density of 4 × 10^4^ cells per well in differentiating medium. The cell growth curve was assessed on the basis of cells number counted using a Burker’s camera. Subsequently, the adherent cells were enzymatically detached from the surface of wells using 1 mM EDTA and 0.25% trypsin and the cell number was determined in a counting chamber.

An XTT (Sigma-Aldrich) assay was used to examine the viability of cells according to the manufacturer’s protocol. Cell cultures were washed three times with PBS, and then XTT working solution was added and left for 4 h at 37 °C. The final product of the reaction was measured using a FLUOstar OPTIMA ELISA plate reader (BMG LABTECH GmbH) at 450 nm. The cell number and viability on days 1, 7, 14 and 21 of culture was determined. Seven independent experiments were performed. The data are presented as means ± standard deviation. Student’s *t*-test was used for statistical analysis to compare the results. *p* < 0.05 was considered significant.

### Determination of alkaline phosphatase (ALP) activity

The culture medium was removed and the wells were washed three times with PBS, and then lysed for 1 h at 4 °C with cell lysis buffer consisting of 10 mM Tris-HCl (pH 7.5) and 0.1% Triton X-100 (Sigma). Then, 50 μl of the lysates were assayed using p-nitrophenyl phosphate substrate (Sigma) according to the manufacturer’s instructions. The colorimetric reaction was carried out at 37 °C for 30 min, then stopped using 3 M NaOH and EDTA solution. The absorbance of the reaction product was determined at 405 nm using the FLUOstar OPTIMA ELISA reader. The ALP activity (U/cell number) was measured on days 7, 14 and 21. Seven independent experiments were performed. The data are presented as means ± standard deviation. Student’s *t*-test was used for statistical analysis to compare the results. *p* < 0.05 was considered significant.

### Total protein content assay

On days 7, 14 and 21 of culture, the total protein content was determined in the cell lysates using a Protein Assay BCA kit (Pierce Biotechnology, Inc., Rockford, USA). Test.). The test was done according to the manufacturer’s protocol. Absorbance was measured at 562 nm and the total protein content was calculated. Seven independent experiments were performed. The data are presented as means ± standard deviation. Student’s *t*-test was used for statistical analysis to compare the results. *p* < 0.05 was considered significant.

### Determination of collagen production

The collagen assay is based on binding of Sirius red dye to the triple helical collagen fibril. On days 7, 14 and 21 of culture, the cells were washed three times with PBS, and then 0.1% Sirius red in saturated picric acid was added (Sigma-Aldrich), and incubated for 60 min at room temperature. Then the plates were rinsed three times with 10 mM HCl. The presence of collagen was observed under an inverted microscope (Nikon Eclipse TE2000-u). The collagen-binding stain was finally eluted with 0.1 M NaOH and absorbance was read at 540 nm using the FLUOstar OPTIMA ELISA reader. Seven independent experiments were performed. The data are presented as means ± standard deviation. Student’s *t*-test was used for statistical analysis to compare the results. *p* < 0.05 was considered significant.

### Actin microfilaments staining

HBDCs were plated at a density of 2 × 10^4^ cells on a cover slide (1.9 cm^2^) in standard medium. The next day, the cells were treated with differentiating medium. On days 7, 14 and 21 of culture, the attached cells were washed three times with PBS (Gibco), fixed in 3% formaldehyde/PBS for 10 min, and permeabilized in 0.1% Triton X-100/PBS (Sigma) for 1 min at room temperature. The washing step was followed by incubation with TRITC*-*conjugated phalloidin (Sigma-Aldrich) diluted in PBS (1:200) for 40 min, at room temperature. After a final wash in PBS, specimens were mounted in Ultra Cruz Mounting Medium containing DAPI (Santa Cruz Biotechnology, Santa Cruz, CA) and observed using a fluorescent microscope (Nikon Eclipse TE2000-u) connected to Nikon Digital Sight DS-U1 camera. Three independent experiments were performed and representative images are shown.

### Immunofluorescence localization of β1 integrin subunit and FAK

HBCS were seeded at a density of 2 × 10^4^ cells on a cover slide in standard medium. The next day, they were treated with differentiating medium. On days 7, 14 and 21 of culture, the cells were washed in PBS and fixed with 3% formaldehyde in PBS (Gibco). Then, the cells were permeabilized in 0.1% Triton X-100 in PBS, washed three times in PBS and incubated in 0.25% glycine in PBS for 30 min at room temperature. Non-specific binding was blocked with 3% bovine serum albumin (BSA, Sigma) diluted in PBS for 30 min. Cells were incubated with primary antibody overnight at 4 °C. Primary antibodies were mouse monoclonal anti-β1 integrin (Santa Cruz Biotechnology) and mouse monoclonal anti-FAK (Santa Cruz Biotechnology). They were diluted 1:100 in 3% BSA/PBS. Then, the cells were washed in PBS and incubated with secondary biotin-conjugated antibodies (1:100 in 3% BSA/PBS; Santa Cruz Biotechnology), followed by incubation with Extravidin-TRITC (1:100 in 3% BSA/PBS; Sigma-Aldrich). Unlabeled cells and cells labeled with a secondary biotin-conjugated antibody and with extravidin–TRITC were used as a control.

After a final wash in PBS, specimens were mounted in Ultra Cruz Mounting Medium containing DAPI (Santa Cruz Biotechnology). Images of stained cells were captured using a fluorescent microscope (Nikon Eclipse TE2000-u) connected to Nicon Digital Sight DS-U1 camera. Three independent experiments were performed and representative images are shown.

### Semi-quantitative RT-PCR assay

Total RNA was isolated from cells harvested on day 0, with the cells under non-differentiating conditions in the standard medium, and on days 7, 14 and 21, with the cells cultured with differentiating medium, using a High Pure Isolation Kit (Roche Diagnostics U.S. Heagquarters, Indianapolis, USA).). Specific transcripts were amplified using semi-quantitative RT-PCR with 1 μg of total RNA as a template and specific oligonucleotide primers, using a Titan One-Tube RT-PCR Kit (Roche Diagnostics), according to the manufacturer’s instructions. The sequence of the oligonucleotides used in the RT-PCR was determined based on the literature review (Table 1). The oligonucleotides were synthesized by Singen Biotech Sp. z o.o, Wrocław, Poland.

**Table 1:** List of primers used in the RT-PCR analysis

Target gene	Primer sequences	Fragment size (bp)
ALP	F 5’-ACGTGGCTAAGAATGTCATC-3’ R 5’-AGGGCTCCAACGAGATCGAGATCCG-3’	475
COLI	F 5’-AACTGAAAGCTGAATCCTTCCA-3’ R 5-’TGCCCAAGAAACAAAGCTTC-3’	223
GAPDH	F 5’-TCAAGGAAGCTACGGGCA-3’ R 5’-TGGCAGAAATTACACACACACAC-3’	250
β1 integrin subunit	F 5-’AAGGGCGTGTTGGTAGACATT-3’ R 5’-TGACACATCTCACACGTTTGC-3’	424

The obtained cDNA fragments were separated on 2% agarose LE gels (Roche Diagnostic). The gels were stained with ethidium bromide and the optical density (Odu) of bands was analyzed with Gel Doc 2000 software (Bio-Rad, Hercules, CA, USA). To evaluate the expression levels of transcripts in a semi-quantitative manner, the optical densities of the amplified cDNA fragments were compared with the average density of the housekeeping gene GAPDH. One representative result from each of three independent experiments is presented.

## Results

The primary HBDC culture serves as a suitable model to study osteogenesis. For a comprehensive examination of HBDCs in vitro, the cells were cultured under conditions that allow osteoblast differentiation. The following parameters were evaluated: cell number and viability, total protein content, alkaline phosphatase activity, collagenous matrix production, expression of the osteogenic genes ALP and COLI, localization, and expression of the actin cytoskeleton and two proteins engaged in cell adhesion: β1 integrin subunits and FAK.

The cultures obtained from bone explants of seven different patients were evaluated. The HBDCs were analyzed 24 h after cell seeding, i.e., during the adhesion phase, on day 7 of culture, i.e., during the proliferation stage, and on days 14 and 21 of culture, i.e., during the early and late maturation stages.

### Culture of HBDCs

**Fig. 1:**
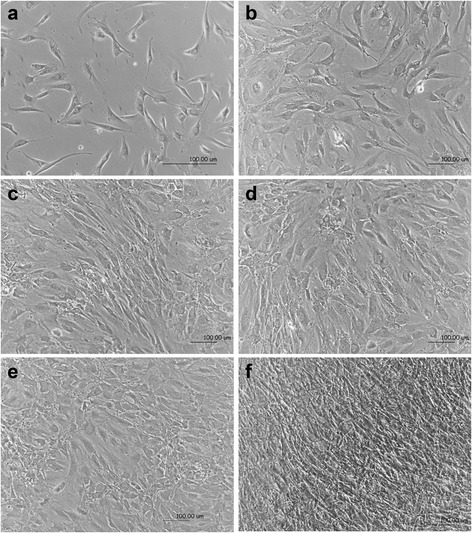
Morphological changes of HBDCs during their in vitro differentiation. The cells were cultured in differentiating medium. Phase contrast images were taken 24 h after cell seeding (**a**), and on day 7 (**b**), 9 (**c**), 14 (**d**), 17 (**e**) and 21 (**f**) of culture. The morphology of HBDCs changed from a fibroblastic-like to a cuboidal shape. Seven independent experiments were performed and representative images are shown. Scale bar: 100 μm

**Fig. 2:**
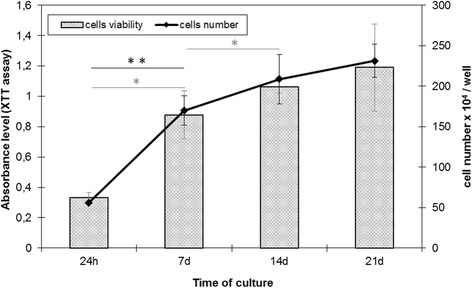
The cell number and viability (XTT assay) 24 h after cell seeding, and on days 7, 14 and 21 of culture. The results of the XTT assay are presented as the absorbance level. It was determined as a linear relationship between cell number and viability. Seven independent experiments were performed. The data are presented as means ± standard deviation. Cell number is in black; cell viability is in grey. Student’s *t*-test was used for statistical analyses. *p < 0.05 (considered significant)

Morphological changes were observed to accurately characterize the primary human osteoblast culture (Fig. 1). Long and narrow fibroblast-like cells were observed during the adhesion (Fig. 1a) and proliferation stages (days 7 and 9, Fig. 1b, c). On days 14 and 17 of culture (Fig. 1d, e), the cell morphology had changed to a cuboidal shape. Finally, on day 21 of culture (Fig. 1f), the cells became elongated. Analysis of the proliferation curve and cell viability showed that HBDCs intensively proliferate during the first 7 days of culture (Fig. 2). Thereafter, as measured on days 14 and 21, the proliferation and cell viability increased at a lower rate (Fig. 2).

### Phenotypic characteristics of HBDCs

**Fig. 3:**
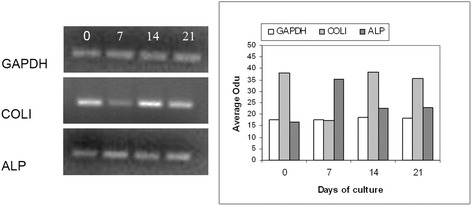
Expression of osteoblast markers, i.e., type I collagen (COLI), alkaline phosphatase (ALP), osteocalcin (OC) determined using semi-quantitative RT-PCR. Analysis of the representative gels and the level of GAPDH, COLI, ALP and OC mRNA (the average optical density of bands; Odu) is presented in the charts (*n* = 3). Results are normalized to the housekeeping gene level (GAPDH). 0 – cell cultured under undifferentiating conditions in standard medium; 7, 14, 21 – cells cultured under osteogenic conditions in differentiating medium

To evaluate the osteoblast phenotype, we used semi-quantitative RT-PCR to investigate the gene expression of the osteoblast markers type I collagen (COLI) and alkaline phosphatase (ALP). Samples were obtained from primary human osteoblasts cultured in undifferentiating conditions (standard medium, point 0) and on days 7, 14 and 21 of culture in differentiating medium. We compared the average density of the GAPDH mRNA band (set to 100%) with the bands corresponding to COLI and ALP mRNAs (Fig. 3) and found that the highest level of mRNA encoding COLI was observed at point 0, when HBDCs were growing in standard medium. On day 7, it was 2.2-fold lower. During osteogenic differentiation, the level of COLI mRNA increased again, and reached the same level as observed at point 0 on day 14, maintaining this level through day 21. During the differentiation of HBDCs the level of ALP mRNA was the highest on day 7, then decreased 1.6-fold and 1.5-fold by days 14 and 21 respectively. There was no difference in the ALP expression level between days 14 and 21.

**Fig. 4:**
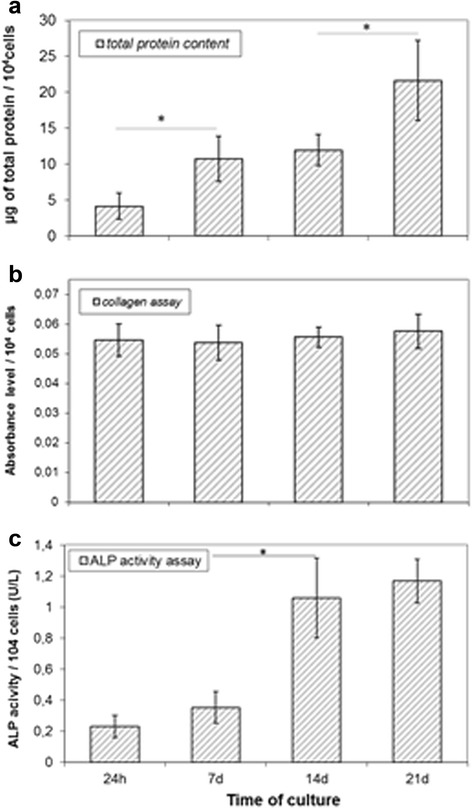
The total protein content (**a**), collagen assay (**b**) and alkaline phosphatase (ALP) activity (**c**). The results of the collagen assay are presented as the absorbance level. The results from seven separate experiments are shown. The data are presented as means ± standard deviation. Student’s *t*-test was used for statistical analyses. *p < 0.05 (considered significant)

**Fig. 5:**
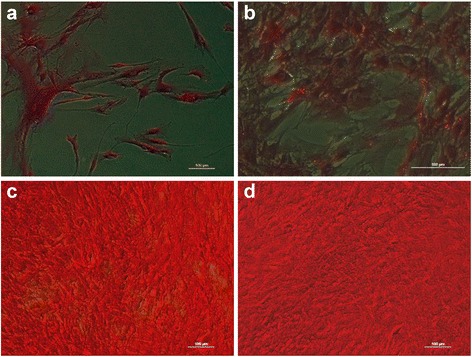
Localization of collagen during differentiation of HBDCs on days 1 (**a**), 7 (**b**), 14 (**c**) and 21 (**d**). Three independent experiments were performed and representative images are shown. Scale bar: 100 μm

The differentiation of human osteoblasts is associated with an increase in total protein synthesis (Fig. 4a), constant production of collagen (Fig. 4b) and an increase in ALP activity (Fig. 4c). At all stages of HBDC differentiation, there was no major difference in collagen production by the cells (Fig. 4b). The results of these analyses precisely reflect those obtained via collagen staining, where all HBDCs in culture showed the presence of collagen (Fig. 5). By contrast, the ALP enzymatic activity increased significantly from day 14 until 21 (maturation stage; Fig. 4c).

### Changes in actin organization and adhesive proteins expression during HBDCs differentiation

**Fig. 6:**
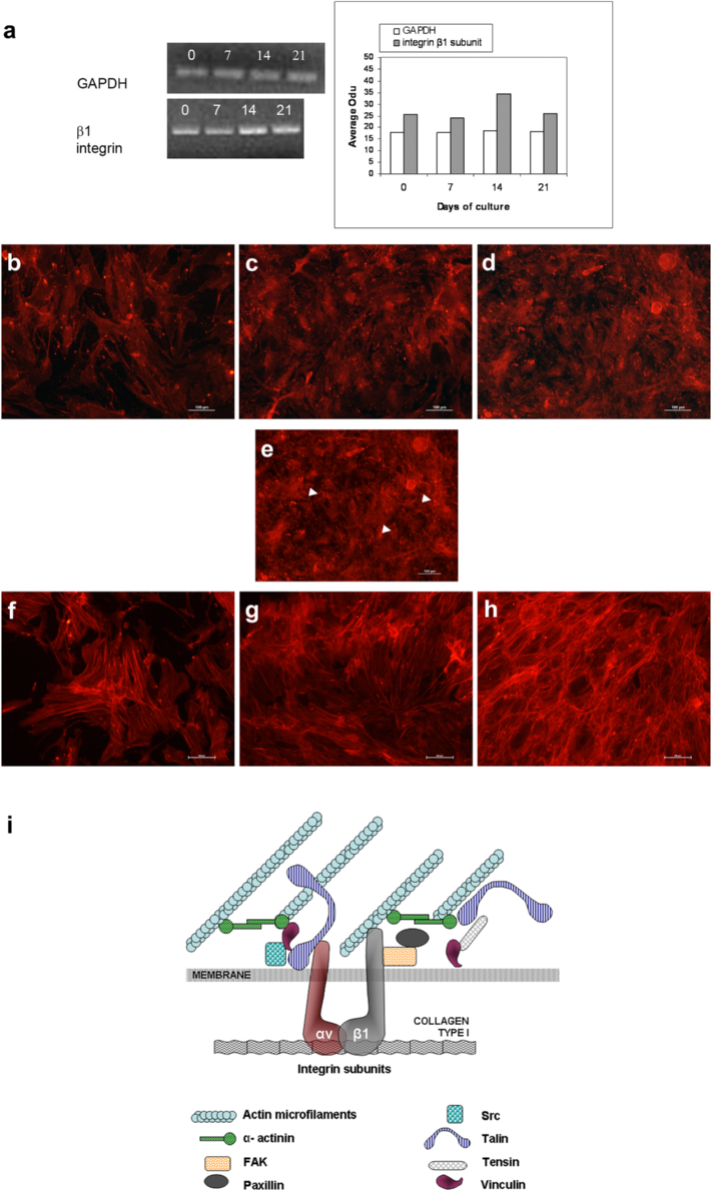
Changes in the expression levels and localization of the β1 integrin subunit during differentiation of HBDCs. **a** Analysis of representative gels. The levels of GAPDH and β1 integrin subunit mRNA are shown in the charts. Results are normalized to the level of the housekeeping gene (GAPDH). 0 – cell cultured under undifferentiating conditions in standard medium; 7, 14, 21 – cells cultured under osteogenic conditions in differentiating medium. **b**, **c**, **d** Immunolocalization of the β1 integrin subunit on days 7 (**b**), 14 (**c**) and 21 (**d**) of HBDC differentiation. Three independent experiments were performed and representative images are shown. Scale bar: 100 μm. **e** Immunofluorescence staining for osteoblasts terminating in focal adhesion contacts as shown by FAK localization (on day 21 of HBDC culture). Focal adhesion plaques indicated by a white arrowhead. Three independent experiments were performed and representative images are shown. Scale bar: 100 μm. **f**–**h** Localization of actin in differentiating HBDCs on days 7 (**f**), 14 (**g**) and 21 (**h**) of culture. Three independent experiments were performed and representative images are shown. Scale bar: 100 μm. **i** Cell adhesion to ECM proteins is mediated through integrin receptors, which are clustered within adhesive cellular structures known as focal adhesion or focal contact plaques. Focal adhesion plaques are supramolecular assemblies containing structural (talin, vinculin, tensin, actin filaments) and signaling components (FAK, Src, paxilin) regulating cell functions via integrins

To answer the question of how cytoskeleton organization and adhesion proteins expression changed during HBDC differentiation, we analyzed FAK, β1 integrin and actin localization in vitro. At first, we analyzed the expression of mRNA encoding β1 integrin subunits (Fig. 6a). The level of the integrin β1 subunit increased during cell differentiation, and its mRNA was the highest on day 14 of culture. The β1 integrin subunit visualized with immunocytochemistry was expressed in undifferentiated adherent cells (Fig. 6b) and in differentiating HBDCs (Fig. 6c, d). The β1 integrin subunit was distributed in the membrane and focal contacts (Fig. 6b–d). Moreover, differentiating HBDCs (day 21) formed numerous focal adhesion contacts, as shown via immunolocalization of FAK, i.e., kinase was engaged in their formation (Fig. 6e).

Actin filaments were stained with phalloidin–TRITC (Fig. 6f–h), and many stress fibers were clearly visible in the cells during proliferation (Fig. 6f, g). The actin filaments were also detected at maturation stage (Fig. 6h). By day 7 of culture, HBDCs had spread to form numerous actin stress fibers, observed as long, linear, well-defined filaments arranged in large bundles tending to run parallel to each other (Fig. 6f). By day 14 of culture, the cell number had increased noticeably, with the cells having cuboidal shape and being substantially less spread. Actin cytoskeleton distribution had also started to change (Fig. 6g). In the last phase of osteoblast differentiation, the concentration of actin was the highest and a complex network of actin filaments was detected (Fig. 6h). Thus, differentiation of HBDCs accompanies reorganization of the actin cytoskeleton.

## Discussion

Human osteogenic cells can be isolated from human bone and are able to differentiate and produce ECM proteins, which are the substrate for mineralization during bone formation. The aim of this study was to characterize the HBDC phenotype and investigate the osteoblast potential of these cells in vitro. Differentiation of HBDCs has been described as a three-step process consisting of the adhesion phase, proliferation phase and osteoblast maturation.

HBDCs are considered a source of cells for a therapy. The characteristics of these phases at molecular level are very important when considering using autologous bone transplants for human bone reconstruction. Isolated osteoblast cells prepared for implantation could be differentiated in vitro [23]. To induce differentiation of isolated HBDCs, a differentiating medium containing vitamin C, vitamin D3 and dexamethasone was used. The role of vitamin C is to stimulate the synthesis of COLI [24]. Dexamethasone is a synthetic glucocorticosteroid that inhibits proliferation and induces differentiation of progenitor cells to osteoblast phenotype [25]. Vitamin D3 and dexamethasone regulate the differentiation of osteoblasts affecting the level of ALP expression and activity [26].

In this study, we used HBDCs from the first passage. As shown by Coelho et al., serial cell passages affect the number of cells and lead to a loss of the osteoblast phenotype [27]. It was noticed that the proliferation and viability of HBDCs first increased and then gradually stopped, starting from day 14 of culture, i.e., when the cells began to differentiate.

Markers such as ALP and COLI were monitored to assess the osteoblast potential of isolated HBDCs. The level of ALP mRNAs is one of the crucial factors to determine osteoblast differentiation. It is known that the expression of ALP mRNA is present in the early stages of osteoblast differentiation, increasing in mature osteoblasts and decreasing when osteoblasts differentiate to osteocytes [28]. Our results showed that ALP mRNA was detected in undifferentiated HBDCs, increased at the proliferation stage and slightly decreased during cell maturation. The time course of ALP gene expression roughly fits with the increase in ALP activity as shown using an osteosarcoma cell line [29]. The increase in ALP activity correlates with the formation of bone-like structures in vitro [30]. Our study showed that ALP activity was detectable 24 h after cell seeding and increased during human osteoblast maturation.

The expression level of osteogenic markers varied depending on the donors, but the trends were the same. The extracellular matrix of the bone is composed of 90% collagenic proteins (type I collagen 97%, type V collagen 3%) and 10% non-collagenic proteins (osteocalcin 20%, osteonectin 20%, bone sialoproteins 12%, proteoglycans 10%, osteopontin, fibronectin, bone morphogenic proteins, etc.). All these proteins are synthesized by osteoblasts and most are involved in cell adhesion. Fibrillar COLI, the predominant organic matrix composite in bone, functions as a three-dimensional template to control the spatial aspect of mineralization. The covalent intermolecular cross-links are the final collagen post-translation modification crucial for the stability of the fibrils. 1,25(OH)_2_D_3_ regulates collagen post-translational modifications and maturation in osteoblast cell culture [31].

We demonstrated that isolated and cultured HBDCs changed their morphology and that the expression and activity of ALP increased. Thus, the cells were able to differentiate in vitro into osteoblasts. However, the level of COLI mRNA and protein, the major component of ECM in bone tissue, did not differ significantly during HBDC differentiation. Nevertheless, as shown previously, the expression of osteocalcin also increased during differentiation of HBDCs cultured under the same conditions [28].

Bone cell differentiation from the undifferentiated state into functional active osteoblasts is a series of steps involving numerous proteins expressed at each stage [32, 33]. Cell adhesion to the substrate is the first phase of the reaction between cells and the extracellular substrate determining the morphology, proliferation and differentiation ability of cells. The oval HBDCs adhere to the surface of a culture dish and change into an elongated shape. This process was connected with reorganization of actin filaments and the formation of focal contacts. The architecture of the actin cytoskeleton is essential to maintain cell shape and signaling leading to proliferation or differentiation in vitro [34]. Also, inhibition of cell proliferation and induction (initiation) of cell differentiation was observed. During this process, HBDCs change shape, actin cytoskeleton reorganization is noticeable and there are an increased number of focal contacts containing FAK and the integrin β1 subunit.

It was previously shown that the αv integrin subunit played an important role in the differentiation of human osteogenic cells [35]. In this study, we focused on other proteins engaged in the formation of focal contacts, i.e., β1 integrin subunits and FAK. Focal contacts are the sites of adhesion between cultured cells and extracellular proteins. Membrane receptors such as integrins play a key role in the formation of focal contacts and link extracellular proteins with the actin cytoskeleton. Numerous proteins like talin, paxillin and vinculin are known to mediate interactions between actin filaments and integrins [36]. Via numerous signaling molecules, integrins connect to the actin cytoskeleton and mediate transduction of signals from the extracellular matrix to the cell nucleus [37]. All bone cell types expressed α1 and α5 subunits, whereas a subpopulation of osteoblast cells expressed α2, αv and β1 integrin subunits [38]. Human osteoblasts cultured in vitro expressed a high level of α1β1, α3β1, α5β1 and αvβ5 and much lower levels of α2β1, α4β1, αvβ1 and αvβ3 integrins.

The main receptor mediating adhesion between osteoblasts and extracellular matrix proteins is the β1 integrin subunit [39]. β1 integrin directly binds a number of structural and regulatory proteins of focal contacts, such as talin [40] and FAK [41]. It was shown that human osteoblasts cultured on substrates containing tripeptide RGD (Arg-Gly-Asp) express the αv and β1 integrin subunits [42]. The RGD motif that is present in several bone matrix proteins is a ligand for integrins and stimulates osteoblast adhesion to the substrate [43]. We previously showed that αv integrin subunit expression significantly increases during HBDC differentiation into osteoblasts [35]. In this study, we demonstrated that expression of the β1 integrin subunit increases during HBDC differentiation. Moreover, both proteins, i.e., the αv [35] and β1 integrin subunits, were localized in numerous focal contacts formed during HBDC differentiation.

## Conclusions

HBDC differentiation is associated with changes in cell shape, the production of collagen and an increase in ALP activity, as well as with the reorganization of actin cytoskeleton and an increased number of focal contacts containing FAK and the integrin β1 subunit. An increase in integrin β1 subunit mRNA expression was also recorded. Thus, we suggest that integrin αv [35] and β1 can be considered as markers of osteogenic differentiation. Moreover, the αv and β1 integrin subunits may be suitable new candidates to evaluate the differentiation of primary human osteoblasts in culture.

## Abbreviations

ALP: Alkaline phosphatase
BSP: Bone sialoprotein
COLI: Collagen type I
ECM: Extracellular matrix proteins
FAK: Focal adhesion kinase
FBS: Fetal bovine serum
HBDCs: Human bone-derived cells
OC: Osteocalcin
ON: Osteonectin
OP: Osteopontin
